# Real-time observations of the impact of COVID-19 on underwater noisea)

**DOI:** 10.1121/10.0001271

**Published:** 2020-05-12

**Authors:** Dugald J. M. Thomson, David R. Barclay

**Affiliations:** Department of Oceanography, Dalhousie University, 1355 Oxford Street, Halifax, Nova Scotia B3H 4R2, Canada

## Abstract

A slowdown in global trade activity due to COVID-19 has led to a reduction in commercial shipping traffic into the Port of Vancouver. The Ocean Networks Canada observatory system provides researchers real-time access to oceanographic data from a wide range of instruments including hydrophones located along the offshore and inshore approaches to Vancouver. Measurements of power spectral density at 100 Hz from four of these bottom mounted hydrophones are presented, along with AIS data and shipping and trade statistics to assess to what extent the economic impact of COVID-19 can be observed acoustically and in near real-time. The quarterly trend in median weekly noise power in the shipping band of frequencies shows that a reduction in noise commensurate with the economic slowdown has been observed at three of the four hydrophone stations.

## I. INTRODUCTION

Marine shipping is a main contributor to ocean noise levels in the 10 Hz − 1 kHz frequency band (Wenz, 1962), with effects on the critical life processes for a wide range of ocean fauna (Popper and Hawkins, 2016). Nowhere is this concern more prevalently felt by both the public and science community than along British Columbia's coastal waters, home to the critically endangered Southern Resident Killer Whales. The link between growth in economic activity and ambient noise levels in the world's oceans has been well established (Frisk, 2012), with much of the 3.3 dB per decade (1950 − 2007) increase attributable to increased commercial shipping (Andrew *et al.*, 2002), a proxy for trade. While the trend of increasing noise levels (Andrew *et al.*, 2011) has slowed or flattened in recent years (Chapman and Price, 2011; Miksis-Olds and Nichols, 2016; Harris *et al.*, 2019), research on the impacts of the human contributions to undersea noise levels on the marine habitat has grown. The deployment of sustained, multi-sensor ocean observatories provides near real-time access to acoustic data, allowing rapid quantification of changing human activities on the environment against a historical baseline. In this study, observations on the Ocean Networks Canada (ONC) Victoria Experimental Network Under the Sea (VENUS) and North-East Pacific Time-series Undersea Networked Experiments (NEPTUNE) arrays provide a first glance at the link between the current novel coronavirus pandemic, reduced economic activity, and underwater sound in the North Pacific Basin and in-shore waters of British Columbia (McVeigh, 2020; Thomson, 2020).

The novel coronavirus, or COVID-19, has had a profound effect on the daily lives of citizens around the world, beginning in China through the end of 2019, and spreading to over 200 countries in subsequent months (Poon and Peiris, 2020). The economic toll of this global pandemic has caused an unprecedented slowdown in global economic activity, an impact that was first apparent in global trade figures for China. In 2019, Canada engaged in almost $100 × 10^9^ (CAD) in trade with China, much of this in the form of exports of raw materials, and imports of consumer and industrial products exchanged by bulk and container shipping through the Port of Vancouver, North America's largest export terminal. With a slowdown in economic activity beginning to appear in trade indicators, a corresponding reduction in ocean noise levels within the shipping noise band may be expected.

The Automatic Identification System (AIS) is a maritime safety communication system that broadcasts a ship's identification, position and other information over radio. This information can be interpreted to estimate economic activity without the delay inherent in official economic data (Arslanalp *et al.*, 2019).

## II. THE NEPTUNE AND VENUS OCEAN OBSERVATORIES

The NEPTUNE and VENUS observatories collect long-time period data (annual to decadal) on the physical, chemical, biological, and geological properties of the ocean, using a wide range of oceanographic sensors deployed at seafloor nodes and on land-based stations. VENUS was the world's first civilian cabled ocean observatory, deployed in 2006 and comprised of a network of nodes in the inland waters between Vancouver Island, the Gulf Islands, the San Juan Islands and mainland British Columbia and Washington State.

The NEPTUNE observatory was brought online in 2009 and spans the Pacific continental shelf with an 800 km cable and nodes placed near shore, on the shelf slope, in the deep sea, and on the Juan de Fuca ridge, where the Juan de Fuca and Pacific continental plates meet.

The data from both observatories, as well as other ocean observations systems in the Arctic, and Atlantic Canadian coast, are served to the public for no cost on the ONC Oceans 2.0 data repository (Ocean Networks Canada, 2020). Four hydrophone stations were logging during the onset of the COVID-19 pandemic and had at least a year of baseline data collected against which to compare. Two stations are located in the Strait of Georgia (SOG), one on the Pacific continental slope, and one near the Juan de Fuca ridge.

### A. Clayoquot Slope and Endeavour nodes, NEPTUNE

The Clayoquot Slope node is located at 48° 41.963′ N, 126° 52.343′ W, 90 km west of Vancouver Island on the continental slope at 1315 m water depth. The node is located ∼25 km north of the Western approaches for international traffic to the Juan de Fuca strait, the gateway the major shipping ports of Vancouver and Seattle. At this site, one hydrophone was operational during the period of interest and had been deployed and recording nearly continuous data since 23 June 2016. The data presented in this manuscript are from the Ocean Sonics icListen HF, (Serial #1353), which was recording with a sample rate of 128 kHz and an acoustic bandwidth of 10 Hz–64 kHz.

The propagation environment on the Clayoquot Slope is characterized by the seasonal variability of oceanographic conditions. In summer months, relatively calm winds and radiative heating lead to stratification of the near-surface layer, where a strong thermocline creates a downward-refracting sound speed profile in the upper 100 m above a weak deep sound channel with an axis near 600 m (Boyer *et al.*, 2013). In the winter, sound speed increases with depth in the surface zone, reaching a maximum near 100 m with a steep negative gradient to a sound channel axis near 500 m. The seafloor is composed of soft muddy sediments up to 5 km thick. Very strong winds and heavy swell prevalent during the winter months heighten the background noise levels recorded at the bottom. The bottom mounted recorder, though not at the axis of the sound channel, does benefit from the shoaling of the wave guide up the slope, allowing a small portion of basin-wide generated sound, such as distant shipping, to arrive at the sensor.

The Endeavour node, at 47° 56.958′ N 129° 05.903′ W, is located on the Juan de Fuca ridge at 2189 m depth and is near the Endeavour hydrothermal vent field. The ridge is at the meeting of the Juan de Fuca and Pacific plates, with the ∼3 km deep Cascadia Basin to the east and the 5 km deep Northeast Pacific Basin to the west. The primary Asia−Pacific Northwest shipping lanes pass 60 km to the north of the node. An icListen HF (Serial #1561) has been deployed at the site from 25 July 2018 until present, recording with an acoustic bandwidth of 10 Hz–32 kHz at a sampling rate of 64 kHz.

The acoustic environment is characterized primarily by a prominent deep sound channel with its main axis near 500 m depth. Up to 1000 m of depth excess is possible in the winter when the surface is cold and well mixed and disappears in the summer when a warm surface layer forms with sound speeds in excess of those found at depth. The result in both cases is a degree of acoustic insulation for the bottom-mounted recorder, with much of the low-frequency acoustic energy trapped in the sound channel above.

### B. Strait of Georgia Central and Eastern Nodes, VENUS

**FIG. 1.**
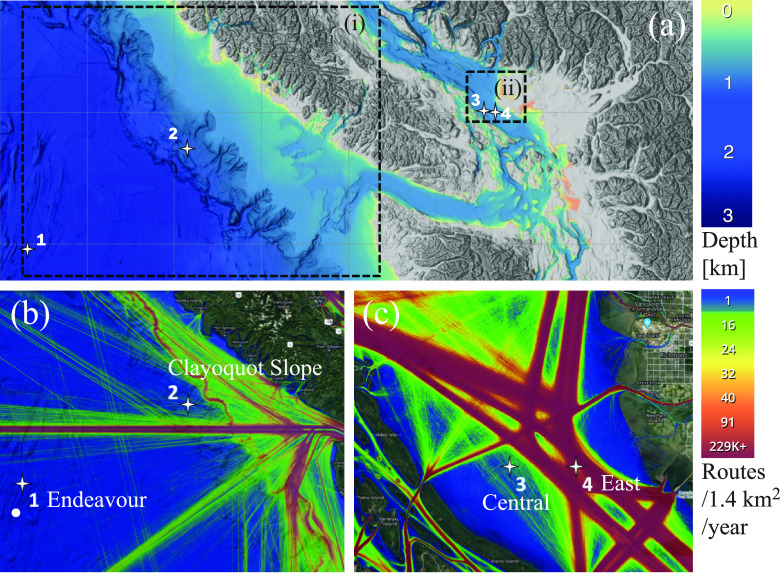
(Color online) (a) Overview of study area bathymetry from NOAA Coastal Relief Model with major shipping routes depicted as AIS total density for 2017 (with AIS data provided by MarineTraffic−Global Ship Tracking Intelligence) shown in (b) for the offshore region (a.i), where the NEPTUNE nodes (1) Endeavour and (2) Clayoquot Slope (white stars) are located, and for (c) the inshore region (a.ii), where the VENUS SOG (3) Central and (4) East nodes (white stars) are located.

Two hydrophone stations within the VENUS network were able to provide near real-time data during the first quarter of 2020, as well as baseline conditions during 2018 and 2019. The SOG Central and East nodes are located outside the port of Vancouver on Canada's west coast, shown in Fig. 1(c).

The SOG Central node is located on the seafloor at a bottom depth of 297 m, at 49° 02.363′ N, 123°25.507′ W, and sits 5 km west of the Duke Point−Tsawwassen ferry route, a car-carrying ferry that runs 16 total crossings using two large (160 m, 10 000 ton displacement) vessels on a typical day. Acoustic recordings were collected using two successively deployed Ocean Sonics icListens deployed, an HF unit (Serial #1350) on the node from 30 September 2018 to 4 March 2020, and an AF unit (Serial #2548) deployed from 5 March 2020 to present. The HF unit records the pressure time series with a sampling rate of 64 kHz and an acoustic bandwidth from 10 Hz–32 kHz, while the AF unit records the pressure with a sampling rate of 32 kHz over the acoustic band 10 Hz–12 kHz.

The SOG East node is located at 49° 02.598′ N, 123° 18.967′ W, approximately 3 km east of the Central node, at a depth of 164 m, and directly under the ferry route. The acoustic data were recorded on an icListen HF (Serial #1560) between 1 October 2018 and 3 March 2020, at which point it was replaced by a JASCO Autonomous Multichannel Acoustic Recorder (AMAR) equipped with four GeoSpectrum M36 hydrophones. The AMAR records with a sampling rate of 128 kHz and an acoustic bandwidth of 5 Hz–64 kHz.

The propagation environment is a typical shallow water Pekeris waveguide, driven by seasonal changes in the vertical sea temperature profile in the near-surface region, with radiative heating and reduced mixing leading to a downwards refracting sound speed profile in the summer months; storm-driven mixing and cold air leading to an upward-refracting sound channel caused by a near-surface thermocline during the winter months; and, transitionary constant sound speed profiles typical in the spring and fall. Seasonal effects are concentrated in the upper 50 m but would be expected to contribute to increased sound levels measured at the bottom-mounted receiver during the summer months, when more sound is refracted towards the sea floor, comprised mainly of silt and sand sediments from the Fraser River.

### C. Acoustic data processing

At all nodes, the hydrophone's internal data acquisition unit logs a pressure time series and passes 5-min files to the node which transmits and stores them on the ONC data servers, where further data products are pre-computed or computed on-demand and delivered to researchers. The acoustic data may be processed and retrieved from the ONC Oceans 2.0 server as the pressure time series in 5-min segments, the 5-min or daily mean power spectra, and the spectral probability density computed daily, weekly, monthly, yearly, or over a user-defined time period.

The data shown in this study were retrieved as weekly spectral probability densities, pre-computed by ONC as follows. The spectra are computed on a 1 s (SOG Central, #2548) or 0.1 s (all others) record length, T, using a single 32 000 point fast-Fourier transform (FFT) with Hann windowing, giving a frequency cell width df = 1/T, of 1 Hz (#2548) or 10 Hz (all others). A known frequency dependent calibration for each sensor is applied to the power spectrum and the 1-min ensemble average of the 60 (#2548) or 600 non-overlapped spectral estimates is computed. Once the 10 080 power spectra recorded during each weekly period have been calculated, the frequency of occurrence of a given power density in a 0.1 dB ref 1 μPa^2^/Hz sized bin, over a given frequency bin size is computed, producing an empirically derived probability density of the power spectral density, referred to as the spectral probability density (Merchant *et al.*, 2013). The frequency bin size is pre-determined and not related to the frequency resolution of the FFT, and ranges from 5 Hz (SOG Central, #5248), 10 Hz (SOG Central, #1350, SOG East, #1560, Endeavour), and 20 Hz (SOG East, AMAR, Clayoquot Slope). Last, the weekly power spectral density median and 1st, 5th, 95th, and 99th percentiles are computed, and the data are packaged for download along with appropriate metadata.

The multi-year time series of median and 1st percentile weekly power at 100 Hz were extracted from the retrieved data and plotted for interannual comparison. The 100 Hz band was chosen as it is well within the band of ship generated noise, while remaining above the typical flow (pseudo) noise band for the three environments considered, and low enough to minimize the contribution of local wind wave noise the sound field. The weekly time resolution was used to smooth the daily variability caused by the scheduling of ships, while providing enough data points to assess changes in the sound field over the relatively short time period of January−April, 2020 during which the expected change occurred.

Both SOG hydrophones were replaced during a maintenance cruise through the first week of March 2020, compromising the validity of direct comparison of median weekly power levels. Due to either sensor drift, or calibration errors, a ∼10 dB increase in the median daily power was observed in the SOG East node data, and a ∼10 dB decrease in the same metric was seen in the SOG Central data, making comparisons of absolute mean power level impossible.

In order to compare data across this time period, the weekly change in median power at 100 Hz, Δ (in units of dB/week), is computed. For each quarter in the calendar year, dΔ/dt, the linear slope of Δ and Δ_0_ the intercept, are computed using an ordinary least-squares linear fit, where the data point between the last week of February and first week of March is ignored. Last, the mean weekly change in median power, ⟨Δ⟩, is computed over each entire quarter, from the fourth quarter in 2018 to the first quarter in 2020. The slope of Δ, which has units of dB/week^2^, is analogous to acceleration and describes the speed at which the underwater sound field is becoming louder or quieter, while the intercept is analogous to the initial velocity. ⟨Δ⟩, analogous to the mean velocity, provides a measure of the average weekly increase or decrease in noise power over an entire quarter. As with the kinematic equations of motion, these values can be integrated over time appropriately to estimate the change in median weekly power spectral density at 100 Hz using

$$X_{q}=\langle \Delta \rangle t,$$ (1)

where *X* is the quarterly change in noise power, *t* is time in weeks, and dΔ/dt is assumed to be zero, or using

$$X_{q}=\Delta_{0}t+\frac{1}{2}\Delta t^{2},$$ (2)

where a constant dΔ/dt is assumed.

## III. DATA AND RESULTS

### A. Trade activity and Automated Identification System, Strait of Georgia

**FIG. 2.**
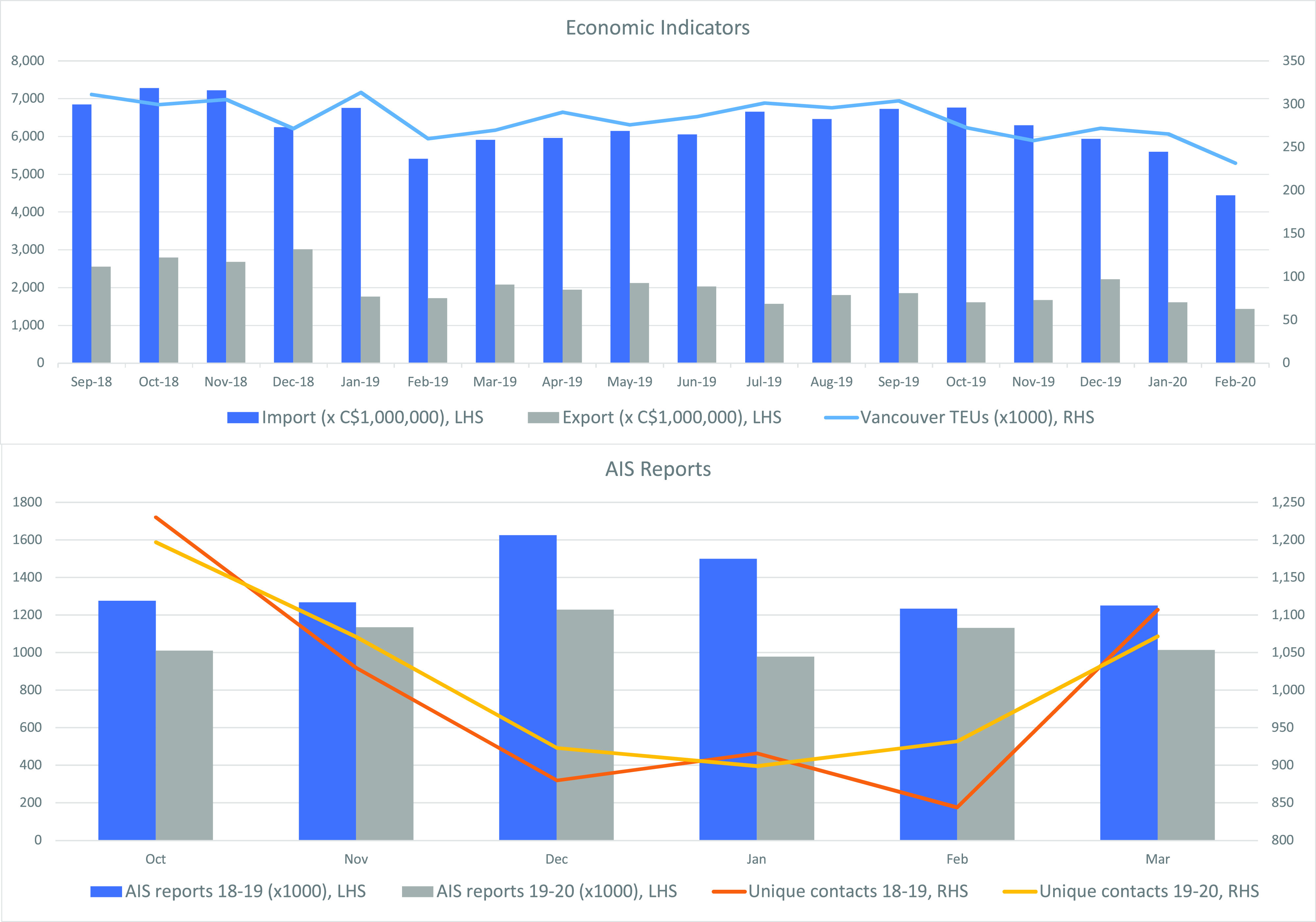
(Color online) Economic indicators (top) for the period 18 September–20 February, with total import and export of tradeable merchandise between Canada and China (LHS), (Statistics Canada. 2020, Table XII-10-0119-01), and 20-foot equivalent unit containers shipped through the Port of Vancouver (RHS). AIS reports for the periods 18 October–19 March and 19 October–20 March (bottom), with total number of AIS contact reports.

Statistics Canada releases monthly Canadian International Merchandise Trade data based on customs records submitted for all imports and exports with a 1-month lag. As shown in Fig. 2, these data typically range between CAD 5–7 × 10^9^ of imports and 1.5–3 × 10^9^ of imports; in February 2020, both measures reached 18-month lows of 4.4 and 1.4 × 10^9^, representing a 16.3% overall reduction in import (−17.5%) and export (−12.4%) activity for the first 2 months of 2020 compared to 2019 (Statistics Canada, 2020).

The Port of Vancouver tabulates the tonnage of cargo shipped through the port and reports it in a monthly report with a 1-month lag. At the end of February, 2020, the port saw a 13.3% overall reduction in import (−12.2%) and export (−14.5%) of 20-ft equivalent unit (TEU) container activity (Port of Vancouver, 2020) for the first 2 months of 2020 compared to 2019. The effect of this trade slowdown may be a reduced number of bulk cargo carrier trips and vessels travelling with lighter than usual loads, both of which would have an impact on the underwater noise field.

AIS data were obtained from exactEarth, a Canadian space-based data service provider which operates a nine-microsatellite constellation to provide global AIS coverage, within a 1600 km^2^ box outside the Port of Vancouver. AIS data were collected for two 6-month periods starting in Oct. 2018 and Oct. 2019 and processed to convert raw-format messages into tables identifying the ship by its unique MMSI number, message time and ship position. Pre-processed AIS data were sorted to count the total number of AIS message broadcasts within the area as well as unique MMSI numbers reporting in each period (Metcalfe *et al.*, 2018). AIS data show a 21.5% reduction in the total number of AIS reports for the first 90 days of 2020 as compared to 2019; the number of unique contacts reporting in each monthly period increased by 1% in the first 3 months of 2020 as compared to 2019.

### B. Acoustic data, Endeavour and Clayoquot Slope

**FIG. 3.**
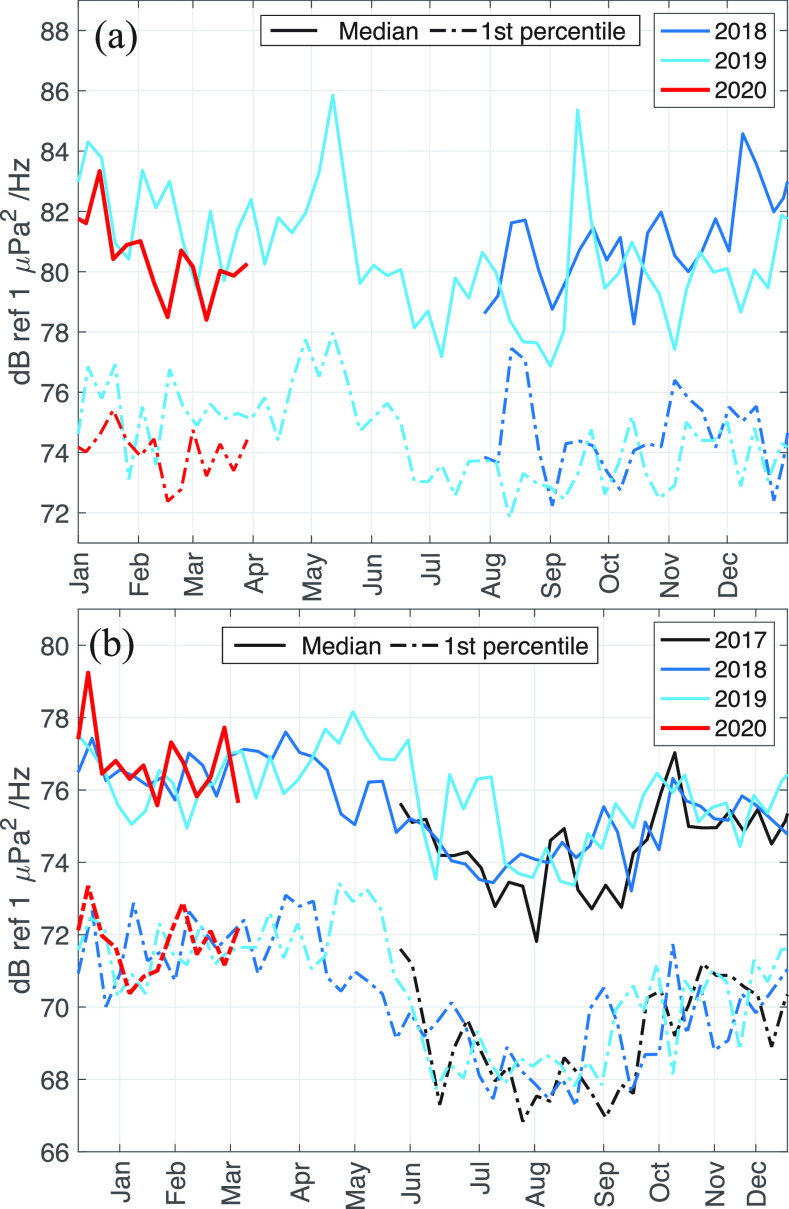
(Color online) Yearly time-series of median (solid line) and 1st percentile (dashed-dotted line) weekly power spectral density at 100 Hz recorded at (a) the Endeavour and (b) the Clayoquot Slope nodes of the NEPTUNE array, for 2017 (black), 2018 (navy), 2019 (blue), and 2020 (red).

At the two deep water sites, the median and 1st percentile weekly power spectral density at 100 Hz were compared on an interannual basis, as shown in Fig. 3. The median power level captures changes in the underwater noise field equally, including changes in the occurrence and magnitude of local, occasional sources, such as passing ships, as well as differences in the persistent background noise, such as distant shipping and wind noise in this low frequency band, while the 1st percentile has a sensitivity to changes of only the quietest 1-min periods, which will be dominated by persistent background noise.

Ambient noise at the Endeavour and Clayoquot Slope nodes show a seasonal variability, with the fall and winter storms leading to elevated sound power levels at the bottom-mounted receivers. The median power levels are approximately 5 dB lower at the Clayoquot Slope station than at the Endeavour station.

At the Endeavour site, the median and 1st percentile power spectral densities at 100 Hz are consistently lower over the period of December 2019−1 April 2020 compared to the same time period from the previous year, shown in Fig. 3(a). During the period August−December, the interannual comparison shows no difference, suggesting that the observed decrease in current noise level is due to a systematic reduction of ship noise in the region, as opposed to natural background noise variability. This is due to a reduction in traffic in the primary shipping lanes linking Asia to the Pacific Northwest of the United States of America and Western Canada, which pass only 60 km north of this station, as discussed in Sec. III A. The mean difference in weekly median power spectral density at 100 Hz over the period 1 January–1 April 2020 compared to the same period one year earlier is −1.5 +/− 0.1 dB. The same metric for the 1st percentile power is also −1.4 +/− 0.1 dB.

The year-over-year change in noise power is negative across the frequency band 10 to 150 Hz, at which point the interannual noise power difference becomes positive, with a value +2 dB over the band 200 Hz–25 kHz. This suggests surface wave action, and thus wind-wave generated noise, is greater in 2020 than during the first quarter of 2019. This observation is confirmed by comparing wave height records from the U.S. National Data Buoy Center station 46 005, which shows a year-over-year quarterly mean significant wave height increase of 0.5 m (US DOC/NOAA/NWS/NDBC National Data Buoy Center, 1971).

The median power levels at the Clayoquot Slope station show no difference when comparing January−March 2020 to the same period in 2019 and 2018, suggesting that vessel activity in the region has not decreased enough to impact the mean weekly sound field. Similarly, the 1st percentile power is equally unchanged in the year-over-year comparison, indicating that either the background noise due to distant shipping in the Pacific basin has not changed, or that the bottom-mounted sensor is too far below the sound channel axis to be sensitive to basin-wide changes. Comparing the absolute sound levels between the two deep water nodes suggests that, though the Clayoquot node is closer in distance to the shipping lanes, the propagation conditions are less favorable or the sensor is in a shadow of the slope bathymetry, resulting in a year-round lower noise levels to be approximately 4 − 6 dB lower at 100 Hz.

### C. Acoustic data, Strait of Georgia

**FIG. 4.**
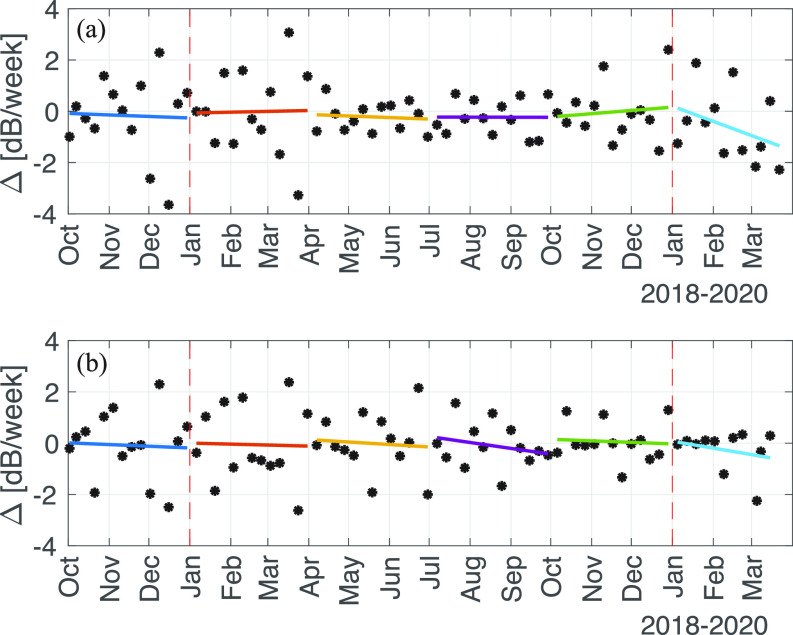
(Color online) Time-series of the change in weekly median power spectral density in dB/week at 100 Hz recorded on the VENUS Strait of Georgia (a) East and (b) Central nodes between October 2018 and April 2020, with the quarterly best-fit line (solid) plotted with a different color each quarter for clarity. The dashed red lines indicate the beginning of each calendar year.

Due to the replacement of sensors in early March, the comparison of year-over-year mean power levels could not be carried out. Instead, the time series of the weekly difference in mean weekly median power spectral density at 100 Hz was investigated at both SOG stations, shown in Fig. 4. At the Central node, the slopes of the best fit lines to the week-over-week differences in median power, Δ, are all less than 0.03 dB/week^2^ in magnitude, and absolute value of the quarterly mean changes are less than 0.2 dB/week during the last quarter of 2018 and all four quarters of 2019. In the first quarter of 2020, the mean weekly rate of change is –0.59 dB/week, while the slope of the best fit line is −0.12 dB/week^2^. These two values give a quarterly change in the power spectral density at 100 Hz of –7.1 dB by Eq. (1) and –5.8 dB by Eq. (2).

It should be noted here that the discussion of error has been limited due to the low number of samples (13) used in the estimates the unknown parameters in the linear least squares fit. The mix of noisy processes (meteorological, oceanographic, ship contact occurrence, and the many factors that determine ship noise source properties) that determine the weekly mean power levels and their weekly difference cause large outliers, producing large estimates of standard error. With the propagation of errors through Eqs. (1) and (2), the estimates gain considerable uncertainty (upwards of 80%−90%), so the figures given above should be interpreted with caution. However, inter-quarterly comparisons show that the observed changes are significant. For example, the next largest estimated quarterly change computed using Eqs. (1) or (2) occurred in 2018, quarter 3 and is of –2.7 dB, while the mean of the absolute quarterly changes over all other quarters except the most recent is only 1.6 dB. Additionally, the value of dΔ/dt in 2020, quarter 1 is over four times greater than the next largest value, 0.03 dB/week^2^ in 2019, quarter 4, and over nine times larger than the mean value.

The observed changes at the Central node are consistent with the observations at the East node, though smaller in magnitude. In the first quarter of 2020, the mean weekly rate of change is –0.25 dB/week, while the slope of the best fit line is −0.06 dB/week^2^, giving estimates of total quarter power spectral density change at 100 Hz of –2.7 and –2.3 dB from Eqs. (1) and (2), respectively. By Eq. (1), the next largest total quarterly power change is during 2018, quarter 1 and is −0.9 dB. The next largest value of dΔ/dt is in 2019, quarter 3, at –0.05 dB/week^2^, but causes little change in quarterly power change due to the relatively large value of Δ_0_.

The difference in results between these two stations may be due to the routes of incoming and outbound vessels to the Port of Vancouver, where paths may pass more closely to the East node than the central node, or due to the large uncertainties in parameter estimates. The on-going collection of data at both nodes will improve the estimates allowing longer averaging times (e.g., monthly and quarterly) with enough data points to reveal trends. The re-calibration of the recently recovered sensors from both SOG nodes may allow the direct comparison of interannual power spectral density, avoiding the current analysis which adds uncertainty through parameter estimation and error propagation.

## IV. CONCLUSIONS

The impact that COVID-19 has had on trade activity between China and Canada has led to a decrease in noise, observed in both the deep ocean and inland waters of Canada's Pacific coast using the near real-time ocean sensing networks NEPTUNE and VENUS. An average reduction of 1.5 dB in year-over-year mean weekly noise power spectral density at 100 Hz was observed from hydrophones at the Endeavour node of the NEPTUNE observatory during the first quarter of 2020, despite a year-over-year increase in mean significant wave height in the region. The decrease in noise level may be attributable to reduced commercial ship traffic in the lanes that pass 60 km north of the node which connect the major ports of Vancouver and Seattle to Asia.

No measurable change was observed in either the median or the 1st percentile of the power spectral density at the Clayoquot Slope node. The reduced year-round noise levels at that site compared to the Endeavour site suggest that the station is not as sensitive to ship noise from the Asia−North America ship lane. The insensitivity of the 1st percentile suggests that either ship noise propagating in the sound channel has not reduced, or that the sensor is too far off the axis to be sensitive to such changes. It is expected that studies using data collected from observatories with sensors that exploit the sound channel, e.g., the Comprehensive Ban Nuclear-Test-Ban Treaty hydrophone stations, may provide additional insights into this observed trend.

In the SOG, week-over-week decreases in noise level were observed on both the Central and East nodes of the VENUS observatory during the first quarter of 2020. The mean change in median weekly power spectral density at 100 Hz was –0.59 dB/week and –0.25 dB/week at the East and Central nodes, respectively. A change in sensors during maintenance activity in March made the direct interannual comparison of mean weekly power impossible at both stations. Using the time series of the difference in median weekly power, the total change in power at 100 Hz over the first quarter of 2020 was estimated to be –7.1 and –2.7 dB at the East and Central nodes, respectively. Using a best fit linear approximation to the time varying difference in median weekly power, the same estimates were found as –5.8 and –2.3 dB, though with large uncertainties arising from the fitting process. Both methods produce large negative trend estimates for noise level in the SOG during the first quarter of 2020, while the same method applied to data collected in 2018 and 2019 showed little or no change in trend over each quarterly period. The observed changes coincide with a 16.3% reduction in Canada−China trade activity, a 13.3% reduction in container traffic through the Port of Vancouver, and a 21.5% reduction in AIS transmissions as compared to the same three-month period in 2019, reflecting the considerable drop in commercial ship traffic over this period.

This quick-look study demonstrates the ability of bottom mounted near real-time ocean observatories to rapidly characterize changes in the underwater noise field. The collection of year-over-year baseline data is invaluable for understanding impacts of human activity on underwater sound. Data collected on permanent observatories provide rapid and accessible results for studies on the acoustic health of the oceans, and potential impacts of human generated sound on the marine ecosystem.
